# 

**Published:** 1888-07

**Authors:** 


### NOTICE.

ATLANTA, GA., June 20, 1888.

The partnership heretofore existing between H. V. M. Miller, J. P. Harrison, and A. B. Ashworth, (successor to James A. Gray) under the name and style of the ATLANTA MEDICAL AND SURGICAL JOURNAL, is this day dissolved by the withdrawal of J. P. Harrison.

The undersigned, each owning a one-half interest, will continue the business of THE JOURNAL under the same name and style, and have assumed all its assets and liabilities.

H. V. M. MILLER,
A. B. ASHWORTH.

